# Visual Search in Naturalistic Scenes Reveals Impaired Cognitive Processing Speed in Multiple Sclerosis

**DOI:** 10.3389/fneur.2022.838178

**Published:** 2022-02-14

**Authors:** Johannes Gehrig, Heinrich Johannes Bergmann, Laura Fadai, Dilara Soydaş, Christian Buschenlange, Marcus J. Naumer, Jochen Kaiser, Stefan Frisch, Marion Behrens, Christian Foerch, Yavor Yalachkov

**Affiliations:** ^1^Department of Neurology, University Hospital Frankfurt, Frankfurt, Germany; ^2^Institute of Medical Psychology, Goethe-University, Frankfurt, Germany; ^3^Institute of Psychology, Goethe-University, Frankfurt, Germany; ^4^Department of Gerontopsychiatry, Psychosomatic Medicine, and Psychotherapy, Pfalzklinikum, Klingenmünster, Germany

**Keywords:** multiple sclerosis, eye tracking (ET), cognitive impairment (CI), cognition, visual search (VS), everyday tasks

## Abstract

**Background:**

Standardized neuropsychological testing serves to quantify cognitive impairment in multiple sclerosis (MS) patients. However, the exact mechanism underlying the translation of cognitive dysfunction into difficulties in everyday tasks has remained unclear. To answer this question, we tested if MS patients with intact vs. impaired information processing speed measured by the Symbol Digit Modalities Test (SDMT) differ in their visual search behavior during ecologically valid tasks reflecting everyday activities.

**Methods:**

Forty-three patients with relapsing-remitting MS enrolled in an eye-tracking experiment consisting of a visual search task with naturalistic images. Patients were grouped into “impaired” and “unimpaired” according to their SDMT performance. Reaction time, accuracy and eye-tracking parameters were measured.

**Results:**

The groups did not differ regarding age, gender, and visual acuity. Patients with impaired SDMT (cut-off SDMT-z-score < −1.5) performance needed more time to find and fixate the target (*q* = 0.006). They spent less time fixating the target (*q* = 0.042). Impaired patients had slower reaction times and were less accurate (both *q* = 0.0495) even after controlling for patients' upper extremity function. Exploratory analysis revealed that unimpaired patients had higher accuracy than impaired patients particularly when the announced target was in unexpected location (*p* = 0.037). Correlational analysis suggested that SDMT performance is inversely linked to the time to first fixation of the target only if the announced target was in its expected location (*r* = −0.498, *p* = 0.003 vs. *r* = −0.212, *p* = 0.229).

**Conclusion:**

Dysfunctional visual search behavior may be one of the mechanisms translating cognitive deficits into difficulties in everyday tasks in MS patients. Our results suggest that cognitively impaired patients search their visual environment less efficiently and this is particularly evident when top-down processes have to be employed.

## Introduction

Cognitive deficits are associated with unemployment, fewer social contacts as well as problems with household activities, and thus have an essential impact on the daily lives of multiple sclerosis (MS) patients (1–5). However, it can be rather challenging to understand how poor performance on a neuropsychological test translates into impaired functioning in everyday life if only conventional laboratory measurements with limited relevance to real-life experiences of MS patients are used. Here, we employed eye-tracking analysis during an ecologically valid visual search task to investigate how cognitively impaired MS patients as defined by the diagnostic standard, the Symbol Digit Modalities Test (SDMT) (3, 6), differ from those with a preserved information processing ability.

Eye-tracking has the potential to measure cognition in neurodegenerative diseases (7) and requires only a limited amount of resources. It will become more widespread in the foreseeable future, as the quality of cameras in laptops and tablets and the necessary software has developed to such an extent making it possible to employ them for cognitive experiments, e.g., *via* a web browser (8). Studies using eye-tracking while performing the SDMT have already shown that MS patients differ from healthy individuals, among other measures, with an increased total number of fixations in the test area (9). This suggests that MS patients may have uncertainties during their visual search behavior, possibly reflected by the fact that the target areas are checked multiple times for safety (9). This might be a direct consequence of their cognitive impairment. Similarly, patients with mild cognitive impairment (MCI), often an early stage of the Alzheimer's disease continuum, have been shown to perform significantly worse than healthy controls in a visual search task, which was associated with more pronounced cognitive impairment (10, 11).

However, a typical experimental task using simple visual stimuli (e.g., arrows, dots, etc.) does not reflect a situation from the patients' everyday life. A visual search task with pictures showing everyday situations would possess a much higher ecologically validity. Therefore, we explored eye-tracking during a visual search task with pictures showing everyday scenarios. The decision where and when to move the point of fixation is a key aspect of eye-movement control (12). This decision is driven by visuospatial attention and modulates the speed of visual search (13). In general, attentional top-down control is one of the key aspects in visual search (14) and thus eye-tracking can reflect cognitive processes (7, 9–12).

We hypothesized that MS patients with low SDMT scores would need more time to fixate the location of the target object, as this reflects processing speed and the integrity of top-down processes such as expectations and prior knowledge that determine where to search for a target object. In addition, we expected the less efficient search behavior of cognitively impaired patients to be associated also with a larger amount of fixations of non-target areas prior to the first fixation of the target and a shorter total fixation duration compared to cognitively preserved patients.

## Methods

### Study Population

Forty-three patients with relapsing-remitting multiple sclerosis (RRMS) were enrolled in the study. Four of them did not complete the entire experiment. In addition, three patients were excluded due to a gaze sampling rate below 60% (percentage of correctly measured eye movements by the eye tracker). Data from the remaining 36 RRMS patients were included in the analysis. Table 1 shows the demographic and clinical characteristics of the sample.

**Table 1:** Demographic and clinical characteristics of the patients.

	**SDMT z**	**Unimpaired**	**Impaired**	**Sum of squares**	***F*** **-value**	***p*** **-value**
Age (years)^a^	>-1 vs. < -1	36.29 ± 11.54	42.50 ± 11.33	308.347	2.343	0.135
	>-1.5 vs. < -1.5	36.44 ± 11.23	44.11 ± 11.77	396.750	3.076	0.088
Visual acuity^a^	>-1 vs. < -1	0.82 ± 0.15	0.76 ± 0.16	0.036	1.500	0.229
	>-1.5 vs. < -1.5	0.83 ± 0.15	0.73 ± 0.16	0.062	2.686	0.110
Disease duration (years)^a^	>-1 vs. < -1	5.33 ± 6.12	10.31 ± 9.20	198.204	3.763	0.061
	>-1.5 vs. < -1.5	5.49 ± 6.12	11.49 ± 9.83	243.240	4.737	**0.037**
EDSS^a^	>-1 vs. < -1	1.81 ± 1.08	3.33 ± 1.68	18.503	10.833	**0.002**
	>-1.5 vs. < -1.5	1.91 ± 1.30	3.56 ± 1.33	18.336	10.704	**0.002**
9HPT (seconds)^a^	>-1 vs. < -1	19.79 ± 3.27	29.13 ± 7.82	696.889	25.796	**<0.000**
	>-1.5 vs. < -1.5	20.65 ± 4.50	29.67 ± 8.22	549.002	17.504	**<0.000**
SDMT (z score)^a^	>-1 vs. < -1	−0.08 ± 0.64	−2.03 ± 0.64	30.135	72.791	**<0.000**
	>-1.5 vs. < -1.5	−0.21 ± 0.71	−2.28 ± 0.51	28.871	63.995	**<0.000**
Gender^b^	>-1 vs. < -1	20 f, 4 m	7 f, 5 m	–	–	0.126
	>-1.5 vs. < -1.5	22 f, 5 m	5 f, 4 m	–	–	0.184

*The means ± standard deviations are shown separately for patients with SDMT z-score >-1 (unimpaired, n = 24) and < -1 (impaired, n = 12) and patients with SDMT z-score >-1.5 (unimpaired, n = 27) and < -1.5 (impaired, n = 9)*.

a *One-way analysis of variance with “SDMT performance” as independent factor*,

b *Fisher-Freeman-Halton Exact Test for contingency tables*.

*F, female; m, male; EDSS, expanded disability status scale; SDMT, Symbol Digit Modalities Test. Bold values indicate significant values*.

The Ethics Committee of the Medical Faculty of the Goethe University Frankfurt am Main approved this study and informed consent was obtained from each patient. Patients were recruited through the neurology department of the University Hospital in Frankfurt am Main. MS was diagnosed according to the 2010 revision of the McDonald criteria (15). All patients had a corrected visual acuity above 0.5 assessed with a vision chart. A trained neurologist assessed the “Expanded Disability Status Scale” (EDSS).

### Design and Data Acquisition

The study comprised two sessions, which were conducted on two different days to reduce the effect of fatigue on performance. Demographics and visual acuity were recorded during the first session. One half of the patients completed first the SDMT and on another day, not more than 6 weeks later, the eye-tracking session. The other half of the patients completed the tasks in reversed sequence.

### Eye-Tracking Design

**Figure 1:**
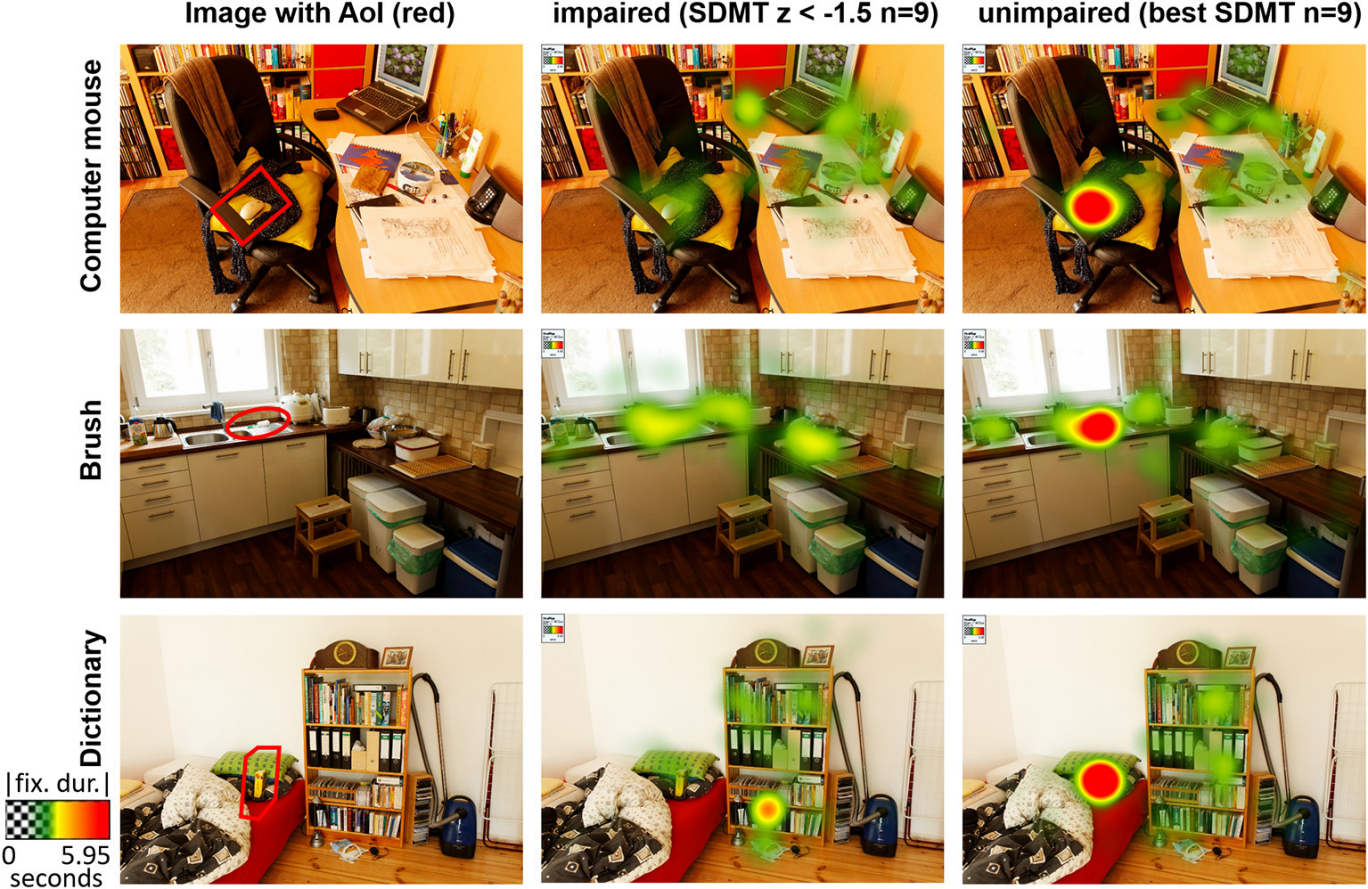
Example pictures of the BOiS-Database (16) showing everyday scenarios. In the left column the area of interest (AoI), which corresponds to the shape of the target object, is marked in red. The heatmap color codes the absolute fixation duration. Heatmaps are shown for the cognitively impaired patients (SDMT *z* <–1.5 (*n* = 9), mean SDMT *z*: −2.28 ± 0.48) and for illustrating purposes the 9 patients with the highest SDMT (mean SDMT *z*: 0.59 ± 0.35).

To investigate the effect of cognitive impairment on visual search in naturalistic scenes, we employed an ecologically valid visual stimulus collection: the BOiS-Database (16) (for examples see Figure 1). The database includes photographs of natural surroundings with scenes from everyday life (e.g., a refrigerator with an open door). In each picture, we defined a prominent object (e.g., a carton of milk, usually expected to be in the refrigerator) as a target. Thirty-five images with a corresponding target object (e.g., milk carton in the fridge door) were chosen. To keep patients alert, we included also 34 images in which the target object was absent and 34 images in which the target was in an unexpected position (e.g., milk carton on the floor). An area-of-interest (AoI) corresponding to the shape (rectangle, circle, ellipse) of the target object was individually defined for each image with a target object. The AoI was defined as an area 2.5 times the size of the target object (Figure 1). Each trial started with the written presentation of the target object's name (e.g., “milk carton”) for 3,000 ms, followed by the presentation of the image for 7,000 ms. The patients were instructed to respond via button press whether the target object was present or absent in the image. Responses were made via a response pad (LogiLink^®^ Keypad).

We used a Tobii Pro X2-60 (Tobii, Danderyd, Sweden) eye-tracker with a 60 Hz sampling rate (binocular) and a maximum total system latency of <35 ms. For each patient a nine-point calibration was performed. The eye tracker with the external processing unit was connected to the recording notebook (Dell Inspirion 7559-0092, Intel^®^ Core™ i7-6700HQ) running Tobii Pro Studio (Version 3.4.8.1348, Tobii, Danderyd, Sweden). The eye tracker was placed on the lower end of the display of the recording notebook. The screen had a resolution of 1.920 × 1.080 pixels and a diagonal of 39.6 cm. We used standardized room lighting.

Tobii Studio was used to calculate the eye tracking parameters of interest in trials where the target object was correctly identified as present. Fixations were calculated using the fixation filter implemented in Tobii Studio. This algorithm assumes that the eyes move between fixation points, and therefore detects fixations when a segment of the eye-tracking signal is constant or changing slowly due to drift, or when there is an abrupt change in the signal indicating that the eyes have moved to a different fixation position. Using those parameters the “time to first fixation” (TFF), “fixations before” (FB), “total fixation duration” (TFD), and “fixation count” (FC) for the AoI were calculated separately for each picture in which the target object was presented. The TFF describes the latency until the expected target position was fixated for the first time. FB gives the number of fixation points before the AoI was fixated the first time. The TFD describes how long the target object was fixated. FC gives the number of fixations on the AoI.

MATLAB (2013b, Natick, Massachusetts: The MathWorks Inc.) was used to prepare the data for further statistical analyses. The mean and standard deviation per patient of the eye-tracking parameters in trials with correct responses were calculated. Additionally the accuracy as a percentage of correct manual responses (button presses) and the corresponding reaction times were calculated.

### Statistical Analysis

#### Sample Size Calculations

Sample size calculations were computed based on the approach described in Hulley et al. (17) and Chow et al. (18) and the website www.sample-size.net. For this purpose, a proportion of 30% cognitively impaired MS patients was assumed (19). Furthermore, we employed the visual search parameter means of MS patients and cognitively healthy individuals as well as the standard deviations reported in Utz et al. (20), who utilized a visual search paradigm and neuropsychological tests to discriminate MS patients from healthy controls. Using the T-statistic and non-centrality parameter, a total sample size of *N* = 35 (*n* = 11 cognitively impaired and *n* = 24 cognitively preserved) subjects was calculated.

#### Neuropsychological Tests

Using age- and education-normative data, SDMT raw scores were transformed to *z*-scores (6). First, the threshold for below-average SDMT performance was set to a conservative and widely accepted threshold for cognitive impairment of *z* < −1.5 (19, 21, 22). Additionally, we performed an exploratory analyses with the aim to determine if eye tracking would detect differences even if the threshold for impairment is less conservative at SDMT *z* < −1. Thus, cognitive impaired patients with a *z* < −1.5 were also in the less conservative group (SDMT < −1).

Baseline demographic and clinical characteristics of the groups (“SDMT *z* > −1 vs. SDMT *z* < −1” and “SDMT *z* > −1.5 vs. SDMT *z* < −1.5”) were compared using one-way ANOVAs and the Fisher-Freeman-Halton Exact Test for contingency (Table 1). Significance was set to *p* < 0.05.

#### Eye Tracking

To evaluate the visual search behavior of patients with MS depending on their cognitive status, we computed one-way ANOVAs with the category “SDMT performance” (“*z* < −1.5 vs. *z* ≥ −1.5” and “*z* < −1 vs. *z* ≥ −1,” respectively), as independent variable, while “time to first fixation” (TFF), “fixations before” (FB), “total fixation duration” (TFD), “fixation count” (FC), and “accuracy” (ACC) were used as dependent variables in each of the ANOVAs. To take into account the significantly different upper extremity motor functions of the two groups, we modeled the average 9-hole peg test (9HPT) performance for the dominant hand as a covariate in the analysis of reaction time. Results for our main analyses (SDMT *z* < −1.5) were corrected for false-discovery rate (FDR) using the Benjamin-Hochberg procedure. Adjusted and corrected for multiple comparisons *q*-values were calculated and significance level was set at *q* < 0.05. The significance for our exploratory analyses (SDMT *z* < −1) was set to *p* < 0.05.

Furthermore, we performed an exploratory analysis where unimpaired (SDMT *z*-score > −1.5) and impaired (SDMT *z*-score < −1.5) MS patients were compared using repeated measurements general linear models with the corresponding eye tracking or performance parameters (TFF, FB, TFD, FC, accuracy, reaction time) as dependent variables, expectedness of the target (expected vs. unexpected position of the target) as within-subjects factor and SDMT performance (unimpaired vs. impaired) as between-subjects factor. Additionally, for reaction time the average 9HPT performance for the dominant hand was included as a covariate. Pearson correlations were computed between SDMT and eye-tracking parameters, accuracy, and reaction times for each of the two conditions expected vs. unexpected. The significance for this exploratory analysis was set to *p* < 0.05.

All statistical analyses were performed using SPSS (IBM Corp. Released 2013. IBM SPSS Statistics for Windows, Version 22.0. Armonk, NY: IBM Corp.).

## Results

When using *z* < −1 as a cut-off for impaired performance in SDMT, impaired and unimpaired MS patients did not differ regarding age, gender, visual acuity and disease duration (all *p* > 0.05, Table 1). The same was found when using *z* < −1.5 as a threshold (all *p* > 0.05, Table 1). However, in this analysis impaired patients exhibited a longer disease duration (*p* = 0.037, Table 1). For both definitions of below-average information processing speed, impaired patients had a higher EDSS value (*p* = 0.002) and 9-hole-peg-test score (*p* < 0.001; Table 1).

Comparing the impaired patients (SDMT *z* < −1.5) with cognitively unimpaired patients we observed significant differences on TFF (*p* = 0.001, *q* = 0.006), TFD (*p* = 0.014, *q* = 0.042), and ACC (*p* = 0.033, *q* = 0.0495). Patients with good SDMT performance were faster in fixating the target for the first time (1.55s ± 0.39s vs. 2.06s ± 0.24s), fixated the target longer (2.38s ± 1.19s vs. 1.28s ± 0.75s) and were more accurate (72 ± 11% vs. 62 ± 10%) in detecting the target (Figure 1, Table 2).

**Table 2:** Visual search behavior depending on SDMT.

	**Unimpaired**	**Impaired**	**Sum of squares**	***F*** **-value**	***p*** **-value**	***q*** **-value**
Time to first fixation (seconds)^a,d^	1.55 ± 0.39	2.06 ± 0.24	1.745	13.559	**0.001**	**0.006**
Fixations before^a,d^	4.44 ± 1.04	4.71 ± 1.44	0.494	0.373	0.546	0.546
Total fixation duration (seconds)^a,d^	2.38 ± 1.19	1.28 ± 0.75	8.021	6.712	**0.014**	**0.042**
Fixation count^a^	5.92 ± 1.53	4.86 ± 2.26	7.534	2.487	0.125	0.15
Accuracy (1 ≜ 100%)^a^	0.72 ± 0.11	0.62 ± 0.10	0.056	4.957	**0.033**	**0.0495**
Reaction time (seconds)^b^	3.43 ± 0.58	3.93 ± 0.61	1.855^c^	5.348	**0.027**	**0.0495**

*Eye-tracking parameters, accuracy and reaction time of patients with SDMT z-score >-1.5 (unimpaired, n = 27) and < -1.5 (impaired, n = 9)*.

a *One-way analysis of variance with “SDMT performance” as independent factor*.

b *Univariate analyses of variance with “reaction time” as dependent variables, “SDMT-performance” as independent variable and average 9HPT performance for the dominant hand as a covariate. The covariate did not reach significance (F-value = 0.790, p-value = 0.381)*.

c *Type III sum of squares*.

d *Reduced number of patients due to missing values (n = 25 vs. 9). Adjusted and corrected for multiple comparisons (FDR) q-values with significance level at q < 0.05. Bold values indicate significant values*.

In the explorative analyses of the visual search behavior of MS patients, we found significant effects of their SDMT performance (SDMT *z* > −1 vs. SDMT *z* < −1) on TFF (*p* = 0.006), TFD (*p* = 0.005), and FC (*p* = 0.033). Patients with average or better SDMT performance were faster in fixating the target for the first time (1.55s ± 0.40s vs. 1.96s ± 0.33s) and they had more (6.08 ± 1.48 vs. 4.71 ± 2.05) and longer (2.47s ± 1.19s vs. 1.29s ± 0.71s) fixations on the target (Table 3).

**Table 3:** Visual search behavior depending on SDMT.

	**Unimpaired**	**Impaired**	**Sum of squares**	***F*** **-value**	***p*** **-value**
Time to first fixation (seconds)^a,d^	1.55 ± 0.40	1.96 ± 0.33	1.236	8.546	**0.006**
Fixations before^a,d^	4.48 ± 1.04	4.59 ± 1.37	0.089	0.067	0.798
Total fixation duration (seconds)^a,d^	2.47 ± 1.19	1.29 ± 0.71	10.280	9.141	**0.005**
Fixation count^a,d^	6.08 ± 1.48	4.71 ± 2.05	14.082	4.985	**0.033**
Accuracy (1 ≜ 100%)^a^	0.71 ± 0.11	0.65 ± 0.11	0.033	2.690	0.110
Reaction time (seconds)^b^	3.44 ± 0.57	3.78 ± 0.68	1.004^c^	2.693	0.110

*Explorative analysis of eye-tracking parameters, accuracy and reaction time of patients with SDMT z-score >-1 (unimpaired, n = 24) and < -1 (impaired, n = 12)*.

a *One-way analysis of variance with “SDMT performance” as independent factor*.

b *Univariate analyses of variance with “reaction time” as dependent variables, “SDMT-performance” as independent variable and average 9HPT performance for the dominant hand as a covariate. The covariate did not reach significance (F-value = 0.444, p-value = 0.510)*.

c *Type III sum of squares*.

d *Reduced number of patients due to missing values (n = 23 vs. 11). Bold values indicate significant values*.

Univariate analyses of variance with “reaction time” as dependent variable, “SDMT performance” as independent variable and the 9HPT as a covariate showed that the main effect of SDMT performance was significant with slower reaction times for the impaired patients (SDMT *z* < −1.5) (*p* = 0.027, *q* = 0.0495, 3.437s ± 0.566s vs. 3.782s ± 0.681s). The 9HPT as a covariate was not significant (*p* = 0.381). Using the explorative cut-off of SDMT *z* < −1 did not show any significant effect on reaction time and the 9HPT performance (*p* = 0.110 and *p* = 0.510).

**Figure 2:**
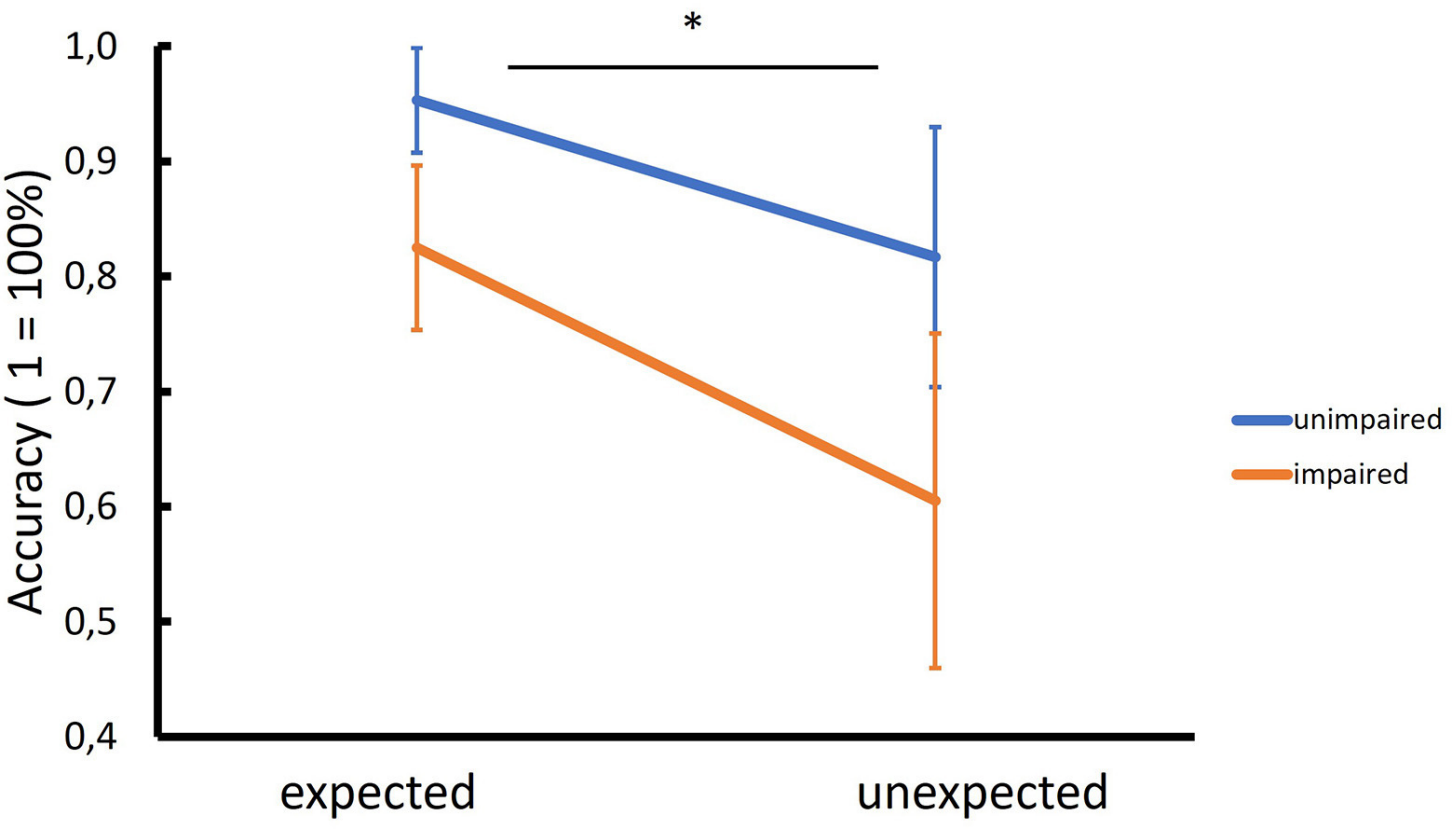
Interaction between cognitive status and expectedness of the location of the target. While cognitively unimpaired MS patients had higher accuracy than impaired patients to detect targets in their expected positions (e.g., milk carton in the fridge), this difference was even more pronounced when the target was in an unexpected position (e.g., milk carton on the floor) (* = significant GLM interaction, *p* = 0.037).

**Figure 3:**
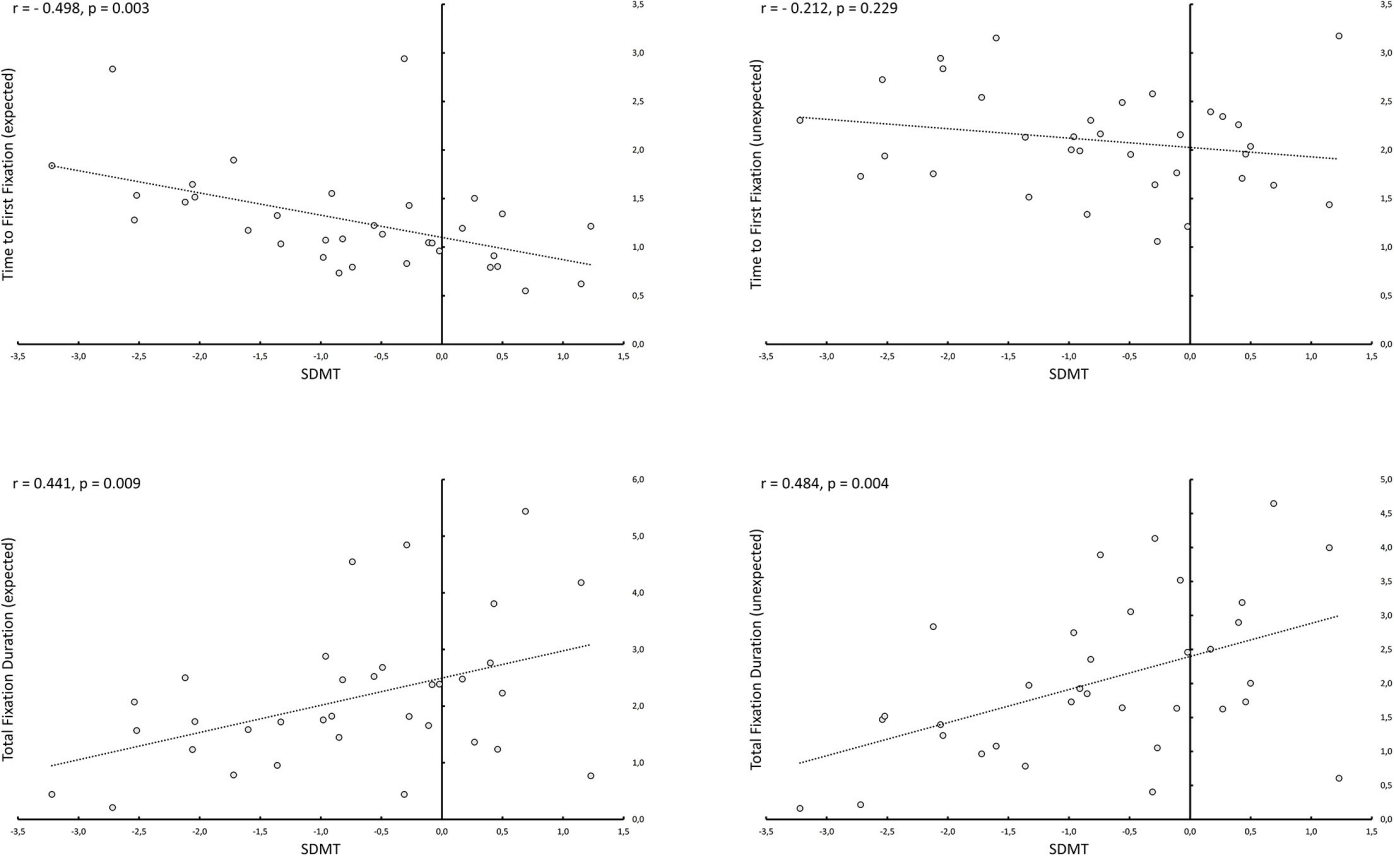
Correlations between SDMT and eye tracking parameters depending on the expectedness of the location of the target. Cognitive performance as measured by SDMT correlated inversely with the time to first fixation for conditions, in which previously announced targets were located in their expected positions (e.g., milk carton in the fridge). However, there was no significant correlation for the conditions, in which the previously announced targets were located in unexpected positions (e.g., milk carton on the floor). For total fixation duration SDMT correlated significantly with eye tracking parameters in both expected and unexpected conditions.

Including the within-subjects factor “expectedness” as another exploratory analysis revealed an interaction between the expectedness of the location of the target and the cognitive status only for accuracy with unimpaired patients being more accurate than impaired patients for the expected condition, while this difference was even more pronounced for the unexpected condition (*p* = 0.037, Table 4, Figure 2). Similar to our primary analysis, we found for TFF, TFD and reaction time but not for FB and FC significant group differences (see Table 4). Not surprisingly, the main effect of expectedness was significant for almost all eye tracking performance and behavioral measures with stimuli in expected locations resulting in better accuracy, shorter reaction times, less TFF and FB as well as more FC (Table 4). We found also significant correlations between SDMT performance and TFF expected (*r* = −0.498, *p* = 0.003, Figure 3) but not TFF unexpected (*r* = −0.212, *p* = 0.229), as well as between SDMT and TFD expected (*r* = 0.441, *p* = 0.009) and TFD unexpected (*r* = 0.484, *p* = 0.004), (see Figure 3).

**Table 4:** Visual search behavior depending on SDMT and expectedness of the location of the target.

	**Unimpaired**	**Impaired**	***p*** **(expectedness)**	***p*** **(SDMT)**	***p*** **(expectedness × SDMT)**
Time to first fixation^a,c^			**<0.001**	**<0.001**	0.67
Expected (seconds)	1.12 ± 0.46	1.69 ± 0.49			
Unexpected (seconds)	1.98 ± 0.47	2.44 ± 0.53			
Fixations before^a,c^			**<0.001**	0.55	0.85
Expected	3.09 ± 0.81	3.42 ± 0.85			
Unexpected	5.80 ± 1.58	6.01 ± 2.37			
Total fixation duration^a,c^			0.19	**0.014**	0.77
Expected (seconds)	2.42 ± 1.28	1.35 ± 0.75			
Unexpected (seconds)	2.33 ± 1.13	1.21 ± 0.79			
Fixation count^a,c^			**<0.001**	0.12	0.51
Expected	6.22 ± 1.69	5.25 ± 2.22			
Unexpected	5.63 ± 1.45	4.46 ± 2.38			
Accuracy (1 ≜ 100%)^a^			**< 0.001**	**< 0.001**	**0.037**
Expected (seconds)	0.95 ± 0.05	0.82 ± 0.07			
Unexpected (seconds)	0.82 ± 0.11	0.60 ± 0.15			
Reaction time (seconds)^b^			**0.03**	**0.003**	0.79
Expected (seconds)	2.05 ± 0.38	2.65 ± 0.50			
Unexpected (seconds)	2.99 ± 0.59	3.63 ± 0.46			

*Eye-tracking parameters, accuracy, and reaction time of patients with SDMT z-score >-1.5 (unimpaired, n = 27) and < -1.5 (impaired, n = 9) across different visual search tasks (target either in expected or unexpected position)*.

a *Exploratory one-way analysis of variance with “SDMT performance” and “expectedness” as independent factors*.

b *Univariate analyses of variance with “reaction time” as dependent variable, “SDMT-performance” and “expectedness” as independent variables and average 9HPT performance for the dominant hand as a covariate. Neither the covariate nor its interactions with the independent variables reached significance (all p > 0.05)*.

c *Reduced number of patients due to missing values (n = 25 vs. 9). Bold values indicate significant values*.

## Discussion

Our findings demonstrate how impaired cognitive processing speed in MS is translated directly into dysfunctional visual search in naturalistic scenes measured with eye-tracking. Impaired patients take longer to find and fixate the target object, and although they take longer, they are less accurate.

They fixated the target object for a shorter time, probably because they first needed more time to locate it in the scene and invested thus more time in exploring the periphery to gain confidence about their decision. The SDMT is diagnostically helpful, but it does not tell us anything about the real world. Our results offer an explanation how impaired cognitive processing limits the patients' daily functioning.

Impaired patients (SDMT *z* < −1.5) indicated with less accuracy whether the searched object was present or not and needed more time to react. This difference was even more pronounced, as demonstrated by the significant GLM interaction for accuracy, when the target was located in an unexpected position (Figure 2). This suggests that visual search in naturalistic scenes is strongly driven by top-down cognitive processes. Our patients were instructed before each trial which object they had to search for. An intact information processing ability would allow the subject to optimize his or her searching strategy (e.g., “milk carton is usually found in the fridge”) and fixate the target more quickly, if it is located in its expected position, as demonstrated by the significant correlation between SDMT performance and time to first fixation for the expected condition (Figure 3). This, however, seems not to be the case, if the target is in an unexpected position (e.g., on the floor)—in this case optimizing the search strategy is not possible by employing top-down cognitive processes, as evident by the absence of correlation between SDMT performance and time to first fixation for the unexpected condition (Figure 3).

Interestingly, when using a more permissive threshold for defining cognitive impairment (SDMT *z* < −1), group differences in visual search behavior remained significant while behavioral measures (i.e., accuracy and reaction time) were no longer significant. This suggests that eye-tracking could be useful in detecting subtle cognitive impairment even before neuropsychological and behavioral performance declines.

Using saccade analysis, previous work has shown that MS patients have a higher error rate (e.g., looking to the wrong side) and a longer latency compared to healthy controls (23). In particular, SDMT and the “Paced Auditory Serial Addition Test” performance correlate with error rate and latency (24). Our study relates to these findings, even though we did not explicitly study saccades. Saccades and fixations are the basis of eye movements (12) and TFF reflects the combined time from all saccades and fixations before the first fixation on the target and thus provides at least an indirectly comparable measure. Thus, our results complement the current literature by employing an ecologically valid paradigm (naturalistic visual scenes) and experimental instructions that are closer to the real challenges MS patients face in their daily lives. Since saccade generation is essential for an optimal performance in a visual search task, the increased error rate found in previous studies using saccade measurements might be linked to our results showing lower accuracy of MS patients. Furthermore, longer latencies in saccade generation might be associated with longer search times (25), which are increased in MS, as we show here using TFF.

It is not surprising that there is a correlation between the SDMT and eye-tracking, as the SDMT assesses visual scanning, memory and perceptual speed, among other functions (26). Pavisian et al. demonstrated that the eye-tracking behavior of MS patients differs from that of healthy individuals during the performance of the SDMT (9). We extend these findings to a more naturalistic task that is directly linked to the patients' daily lives and stress the importance of intact information processing speed for visual search in the context of real-life situations.

With increasing availability of eye-tracking, e.g., based on web browsers, visual screening tests could be performed, for example before an appointment at the doctor's office or even at home. They could be used to identify patients who should undergo a more intensive neuropsychological examination. Furthermore, understanding better how daily functions are affected by impaired information processing could facilitate the development of better cognitive monitoring and rehabilitation strategies. Our results could be explained on the one hand by slowed bottom-up information processing and on the other hand by impaired top-down executive control. Since both perceptual processes and executive control are impaired in MS (27–29), it remains unclear to what extent deficits of bottom-up or top-down processing or their combination are causal for the cognitive deficits of MS patients.

In MS, but also in other neurodegenerative diseases, eye tracking has limitations in cases of severely reduced visual acuity or oculomotor dysfunction (involvement of cranial nerves). The significance of this problem is less essential for patients at the early stages of the disease but might become more serious and hamper implementing eye-tracking in cognitive monitoring or rehabilitation of patients with higher EDSS scores or in advanced progressive stages of the disease.

One further possible limitation is the variable delay between the eye tracking experiment and SDMT testing (up to 6 weeks). In order to prevent fatigue, the two measurements were not performed on the same day, although we knew that this could affect our results. However, since the sequence (SDMT first, eye tracking second and vice versa) of the two measurements was pseudorandomized across all participants, we believe that the influence of any possible systematic bias on our findings has been minimized. Nevertheless, future studies should employ, if possible, a standardized measurement schedule.

Among the limitations of the current study are the small to moderate number of patients included, the absence of progressive MS patients and the cross-sectional nature of our investigation. Longitudinal studies with different disease phenotypes would extend our findings and test the notion that eye-tracking changes predict later clinical and/or cognitive decline. Interestingly, a longitudinal study over 2 years studying antisaccades, has shown that error rate, latency and accuracy worsened in MS patients, whereas EDSS scores remained largely unchanged, which might suggest that eye-tracking could be sensitive to subclinical disease activity and more subtle neurodegenerative processes (30).

Naturalistic cognitive assessments which reflect everyday difficulties that MS patients encounter in their daily routine might be challenging. Visual search tools employing ecologically valid paradigms would be one way to address this problem, but data supporting their validation in MS is limited. On the other hand, there is an increasing amount of data using e.g., cell phone applications or wearables (31, 32) to detect disease progression parameters. After appropriate validation, eye-tracking employed by such applications might prove useful in identifying progression in cognitive impairment.

## Data Availability Statement

The raw data supporting the conclusions of this article will be made available by the authors, without undue reservation.

## Ethics Statement

The studies involving human participants were reviewed and approved by Ethikkommission des Fachbereichs Medizin, Faculty of Medicine, Goethe-University Frankfurt. The patients/participants provided their written informed consent to participate in this study.

## Author Contributions

JG: designed and conceptualized study, analyzed and interpreted the data, and drafted and revised the manuscript for intellectual content. HB and LF: analyzed the data, interpreted the data, and major role in the acquisition of data. DS and CB: analyzed the data and major role in the acquisition of data. MN, JK, SF, and MB: designed and conceptualized study, interpreted the data, and revised the manuscript for intellectual content. CF: designed and conceptualized study and revised the manuscript for intellectual content. YY: design and conceptualized study, analyzed and interpreted the data, and drafted and revised the manuscript for intellectual content. All authors contributed to the article and approved the submitted version.

## Conflict of Interest

YY has been supported by travel grants from Novartis and Sanofi Genzyme, has received an honorarium for active participation in an advisory board by Sanofi Genzyme as well as speaking honoraria by Roche and Sanofi Genzyme. CF reports speaker honoraria and honoraria for participating in advisory boards from Alexion, Bristol Myers Sqibb, Novartis, Teva, Merck, Sanofi-Genzyme, and Roche. CF received research support from Novartis and Sanofi-Genzyme. The remaining authors declare that the research was conducted in the absence of any commercial or financial relationships that could be construed as a potential conflict of interest.

## Publisher's Note

All claims expressed in this article are solely those of the authors and do not necessarily represent those of their affiliated organizations, or those of the publisher, the editors and the reviewers. Any product that may be evaluated in this article, or claim that may be made by its manufacturer, is not guaranteed or endorsed by the publisher.
